# Synthesis of Ni_2_P/CdS and Pt/TiO_2_ nanocomposite for photoreduction of CO_2_ into methanol

**DOI:** 10.1038/s41598-021-87625-w

**Published:** 2021-04-13

**Authors:** Penumaka Nagababu, Sehba Anjum Mumtaz Ahmed, Y. Taraka Prabhu, Ankush kularkar, Subhamoy Bhowmick, Sadhana S. Rayalu

**Affiliations:** 1 Environmental Materials Division, CSIR-National Environmental Engineering Research Institute, Nagpur, 440020 India; 2 Academy of Scientific and Innovative Research (AcSIR), Ghaziabad, Uttar Pradesh 201002 India; 3 Kolkata Zonal Center, CSIR-National Environmental Engineering Research Institute (NEERI), Calcutta, West Bengal 700107 India; 4 Department of Analytical and Structural Chemistry, CSIR-Indian Institute of Chemical Technology, Hyderabad, 500007 India

**Keywords:** Environmental sciences, Environmental social sciences, Chemistry, Energy science and technology, Engineering, Materials science

## Abstract

It is a great challenge to convert thermochemically stable CO_2_ into value-added products such as CH_4_, CH_3_OH, CO via utilizing solar energy. It is also a difficult task to develop an efficient catalyst for the reduction of CO_2_. We have designed and synthesized noble metal-free photocatalytic nanostructure Ni_2_P/CdS and Pt/TiO_2_ for conversion of CO_2_ to methanol in the presence of sacrificial donor triethylamine (TEA) and hydrogen peroxide. The synthesised catalysts physicochemical properties were studied by using several spectroscopic techniques like; XRD, UV-DRS, XPS, TEM, SEM and PL. Quantification of methanol by GC–MS showed encouraging results of 1424.8 and 2843 μmol g^−1^ of catalyst for Pt/TiO_2_ and 5 wt% Ni_2_P/CdS composites, respectively. Thus, Ni_2_P/CdS is a promising catalyst with higher productivity and significant selectivity than in-vogue catalysts.

## Introduction

From the past few decades, substantial changes have been witnessed in the atmosphere due to the burning of fossil fuels^1^ leading to an increase in the demand for renewable energy^2^. Also, the natural resources of fossil fuels are decreasing day by day, thus increasing the demand for an alternate source of energy^3,4^. At present, much of our energy demands are met by fossil fuel, but this resource is not a renewable source and is associated with contemporary environmental issues. Thus, renewable sources of energy shall play a critical role in fulfilling the requirement of energy demand^5^. More efforts have been focused on substituting renewable biomass sources for chemicals and fuels, which possess high energy density and environmentally friendly properties^6^. Mixing alcohol with petroleum products increases the fuel's combustion efficiency due to oxygen and reduces the emission of pollutants into the atmosphere^7,8^. The regular trend for methanol synthesis uses syngas conversion with high pressure and high thermal energy with heterogeneous catalytic reaction over alumina supported Cu/ZnO catalyst^9^. In the petrochemical industry, the production of methanol from CO_2_ reduction an important achievement. In this research area, efforts are continuing to increase the selectivity for methanol by reverse-water–gas-shift (RWGS) reaction in thermal catalytic CO_2_ hydrogenation via Cu/ZnO-based catalyst. The great challenge in this reaction is to maintain the operating temperature and pressure. Due to the competing RWGS reaction, the exothermic nature of methanol synthesis, equilibrium-limited conversion, and the very high activation energy barrier of the CO_2_ hydrogenation to methanol reaction, the process must balance operation temperature megapascal pressures to achieve high selectivity and production rate^10,11^. Fujishima first reported the photocatalysis study in 1972; in recent years, it’s become a common word in chemistry and also various technology products^12^. The theoretical target is to get chemical energy by using light energy with semiconductor materials. The electron present on the conduction band gets activated and migrates to the valance band of the semiconductor with the holes in the conduction band. Thus, the charge carriers facilitate the reaction wherein the electrons react with dissolved CO_2_ present in the reactant stream^13^. The reduction of CO_2_ is mainly depending on the highly reducing electron, which has a high reduction ability to convert CO_2_ into useful methanol^14^. As per Gibb’s free energy values, the CO_2_ and methanol having − 394.4 and − 166.4 kJ/mol energy indicating that the reduction of CO_2_ is an exothermic reaction^15^. Many processes are used for the conversion of CO_2_ into methanol such as (1) chemical conversion method, (2) electrochemical conversion method, (3) photochemical reduction method and (4) photoelectrochemical reduction method (Eq. 1).

1 $$CO_{2} + 3H_{2} \to CH_{3} OH + H_{2} O\quad \Delta {\text{H}}_{{298}} = - 49.51\;{\text{kJ/mol}}$$

Miguel et al.^16^ studied the hydrogenation of CO_2_ to MeOH and CH_4_ which disclosed that thermodynamically CH_4_ formation is more favorable than MeOH generation. Fiordaliso and Diez-ramiez^17^^,^^18^ used Pd_2_Ga and Cu/ZnO catalyst, reported 100% selectivity for MeOH at ambient pressure but these gave a low yield of conversion. TiO_2_ is widely studied as a photocatalyst with a wide bandgap semiconductor (3.2 eV) which can only be excited by UV light. CdS has a narrow bandgap of 2.4 eV and has received considerable interest in the field of water splitting and environmental remediation^19^. It can be a suitable candidate for visible light absorption and fits the thermodynamics requirements for H_2_O/H_2_^20–22^. However, the photocatalytic activity over pure CdS is relatively low due to the instability and recombination of the electron–hole pairs. To overcome the above-mentioned problems, investigators are making a series of approaches that can be applied to improve the photocatalytic activity of CdS and TiO_2_^23–25^. Combining Ni_2_P with CdS has proved to be an efficient way to enhance photocatalytic performance. With this background, we have synthesized two nano-systems Pt/TiO_2_ and Ni_2_P/CdS to convert CO_2_ into methanol in the presence of sacrificial donor triethylamine. The loading Ni_2_P on CdS is also studied by increasing concentration of Ni_2_P from 1 to 5% weight to find the optimum concentration of Ni_2_P. H_2_O_2_ can act as a reducing as well as oxidizing agent depending on pH value. It works as a potent reducing agent in the presence of a basic medium and produces O_2_ and 2H^+^ ions. The present manuscript discusses the significant conversion of CO_2_ to methanol with H_2_O_2_, the selectivity of methanol production (no by-products were observed) and the excellent yield of methanol (2843 μmol/g cat using 5 wt%-Ni_2_P/CdS catalyst). The finding of this has been compared with the conventional catalyst reported in the literature^26^.

## Material and methods

Thiourea (NH_2_CSNH_2_), cadmium nitrate tetrahydrate Cd(NO_3_)_2_·4H_2_O, ethylenediamine, Nickel phosphide (Ni_2_P) and H_2_O_2_ 34%. These chemicals are of analytical grade and used directly without further purification.

### Synthesis of Ni_2_P/CdS

The solvothermal method was used to prepare cadmium sulphide nanorods. 10 mmol and 30 mmol of cadmium nitrate tetrahydrate and thiourea were dissolved in ethylenediamine (30 ml) respectively. These were transferred into 80 ml of Teflon-lined autoclave (stainless steel), which was then kept for 48 h at 160 °C in a hot air oven. After 48 h, an autoclave is set aside to cool at ambient temperature. The yellow colored product is rinsed with deionized (DI-water) water and ethyl alcohol several times. The yellow-colored cadmium sulphide was dried in a vacuum oven at around 60 °C for 6 h^27,28^. Preweighed quantity of nickel phosphide and synthesized cadmium sulphide were dissolved in ethylenediamine. The same autoclave is used to heat the mixture at 140 °C for 12 h. The yellow-green colored product obtained was collected from the cooled autoclave, which was rinsed with ethanol and DI-water several times. The final Ni_2_P/CdS catalyst was dried into a vacuum oven at around 60 °C for 5 h.

### Synthesis of Pt/TiO_2_ nanoparticles

**Scheme 1:**
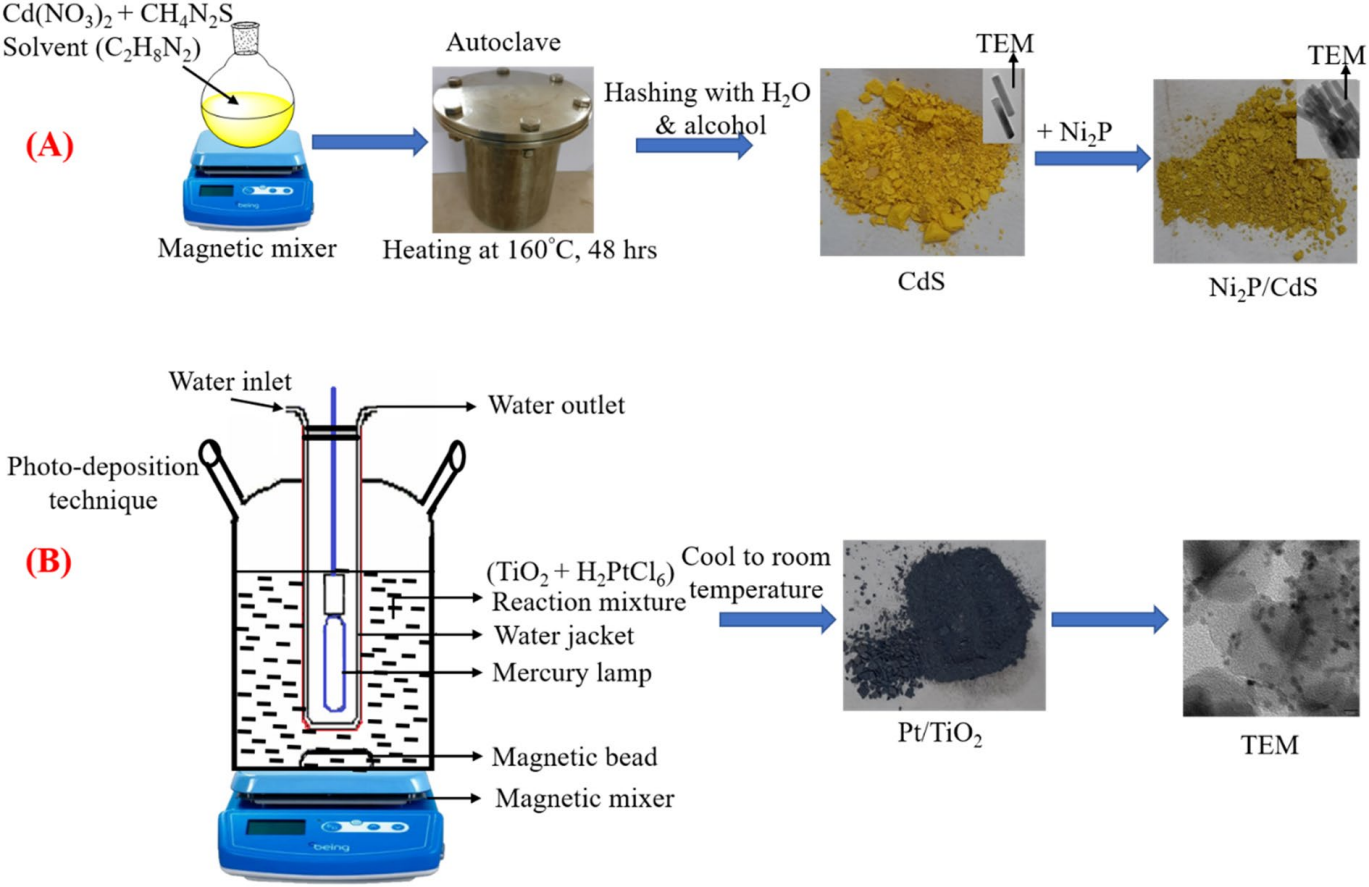
diagrammatic representation of the synthesis of (**A**) Ni_2_P/CdS and (**B**) Pt/TiO_2_.

The photo-deposition technique was used to deposit platinum over titanium dioxide using hydro chloroplatinic acid. The experiment was conducted in a 500 ml irradiation type (inner) reactor. A 350 w medium pressure mercury vapor lamp was used as an irradiation light source and cooled to room temperature with cold circulating water. This photochemical reaction was performed in 400 ml of aqueous reactant solution with 150 mg of titanium dioxide and was continuously stirred magnetically for about 30–45 min to accomplish complete dispersion. Before starting the photo-deposition reaction, nitrogen was purged within a glass reactor to withdraw the air for 25 to 30 min (Scheme 1)^29^.

### Characterization

Morphology of the photocatalysts was determined by scanning electron microscope (SEM) (Tescan, HiPace 10) and HRTEM using JEM-2100F JEOL Japan. The XRD patterns were recorded by using a benchtop X-ray Diffractometer (Model: Rigaku Miniflex II at 30 kV) having a scintillation counter detector. X-ray photoelectron spectroscopy (XPS) analysis has been done by using KRATOS AXIS 165 with Mg Kα irradiation. An Agilent Cary 5000 UV/VIS/NIR spectrophotometer was used to determine the UV/VIS absorption at ambient conditions. Photoluminescence was recorded at Hitachi F-7000 spectrofluorometer. Liquid samples were taken at a different time interval with an airtight syringe and separated by offline GC detected by a flame ionized detector (FID) using helium as carrier gas (Perkin Elmer Clarus 680).

## Results and discussion

### XRD studies

**Figure 1:**
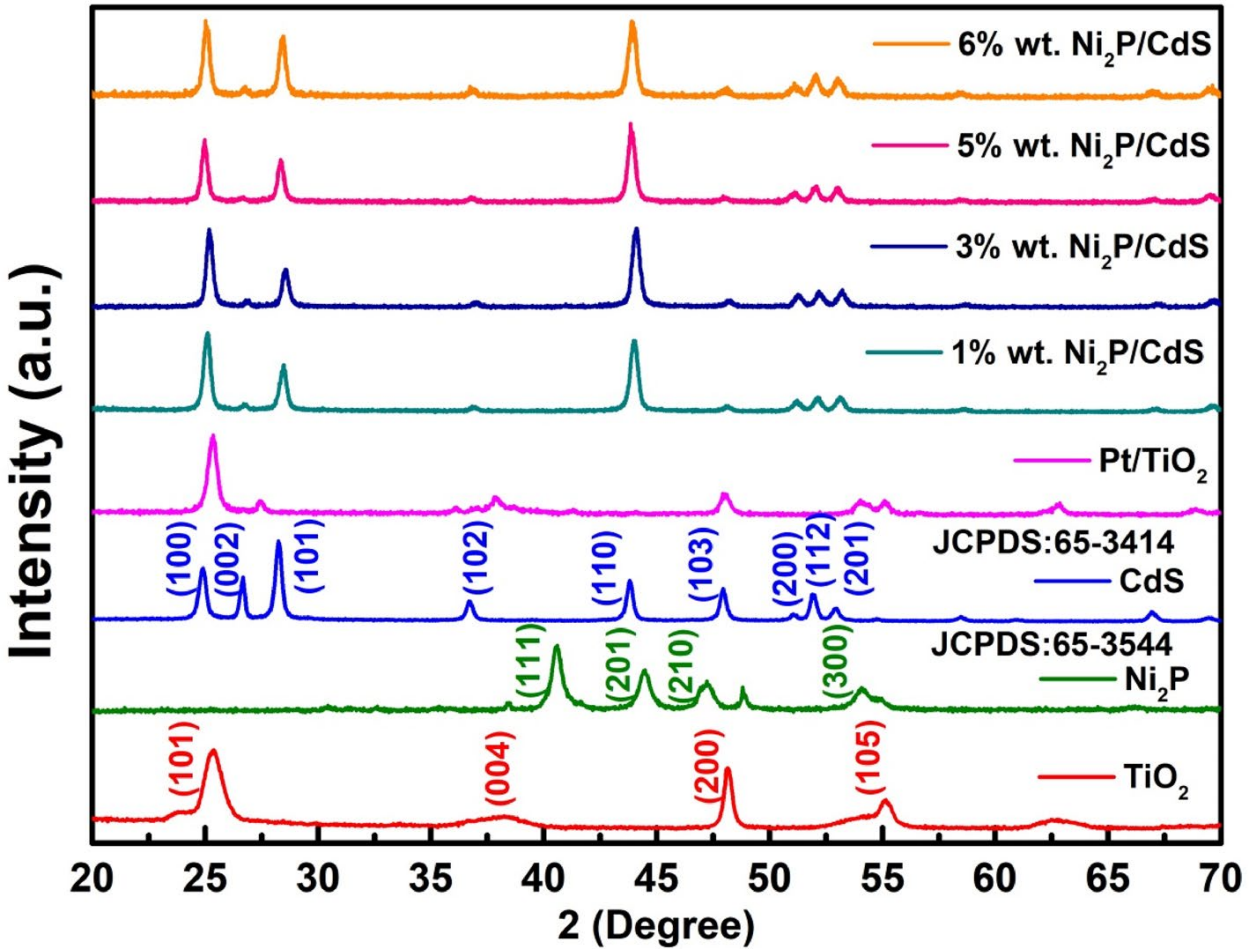
(**A**) XRD patterns of TiO_2_, Ni_2_P, CdS, Pt/TiO_2_ CdS, 1 wt% Ni_2_P/CdS, 3 wt% Ni_2_P/CdS, 5 wt% Ni_2_P/CdS and 6 wt% Ni_2_P/CdS.

**Figure 2:**
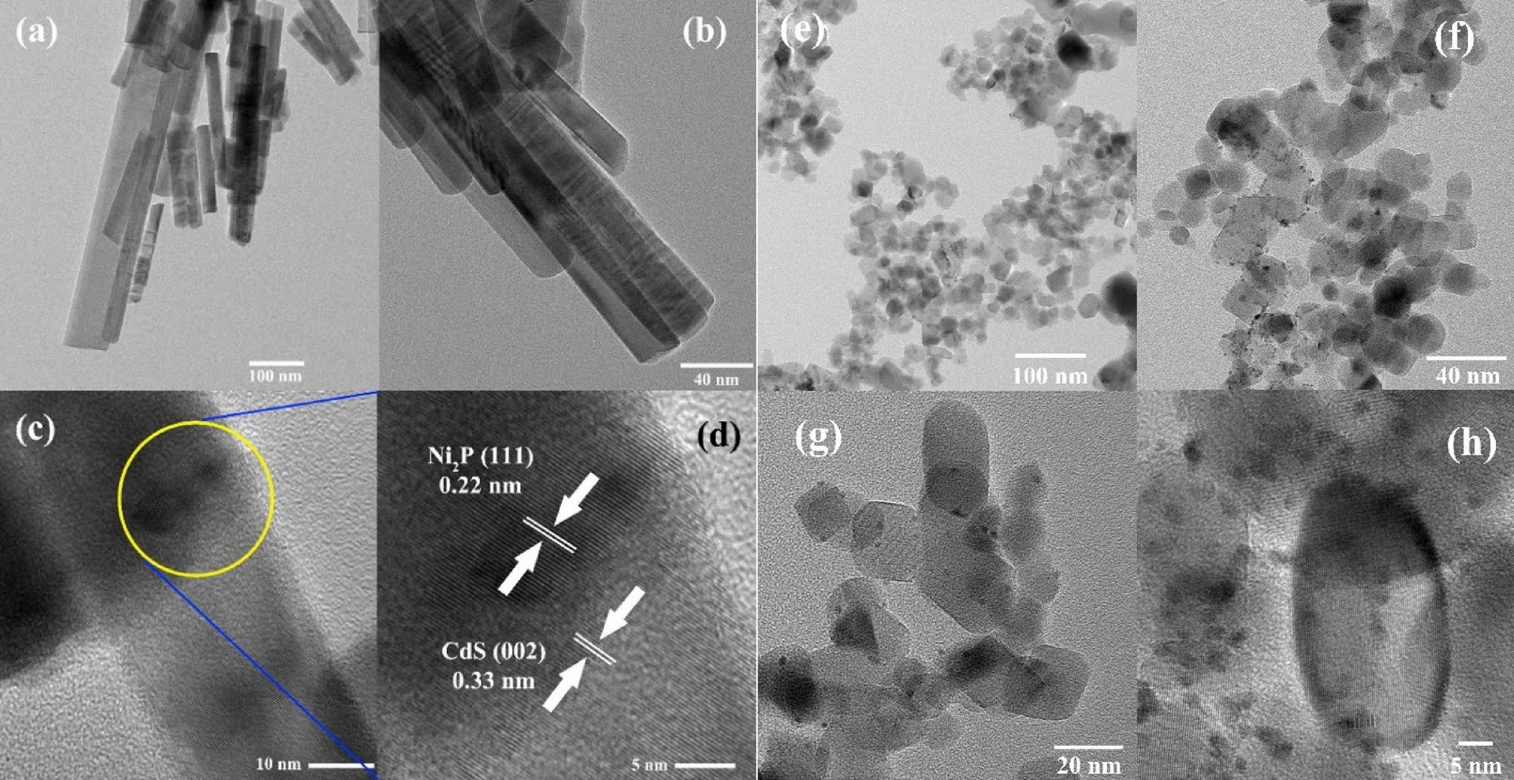
HRTEM images of Ni_2_P/CdS nanorods (**a**–**d**) and Pt/TiO_2_ nanoparticles (**e**–**h**).

The XRD patterns of the TiO_2_, Ni_2_P, CdS, Pt/TiO_2_, CdS, 1 wt% Ni_2_P/CdS, 3  wt% Ni_2_P/CdS, 5  wt% Ni_2_P/CdS and 6  wt% Ni_2_P/CdS are shown in Fig. 1. It is observed that synthesized CdS nanorods have prominent diffraction peaks located at 25.04°, 26.66°, 28.39°, and 43.94° corresponding to the (100), (002), (101) and (110) planes of pure CdS with the hexagonal phase structure (JCPDS file no. 65-3414), respectively, suggesting that CdS has good crystallinity. In the case of Ni_2_P used in the preparation of Ni_2_P/CdS composite diffraction peaks at 40.58°, 44.39°, 47.18°, and 54.08° are observed which matches well with the hexagonal phase standard card of Ni_2_P (JCPDS file no. 65-3544) with (111), (201), (210) and (300) planes, respectively^30^. Besides, the XRD pattern of Ni_2_P/CdS shows prominent diffraction peaks at 25.11°, 26.77°, 28.46°, 36.84°, 43.95°, 48.14° and 51.89° for CdS and Ni_2_P, clearly showing that crystallinity of CdS and Ni_2_P is retained in Ni_2_P/CdS composite^31^. The deposition of Ni_2_P onto CdS shifted the diffraction angle from 43.8° to 43.9°, as indicated in the diffraction patterns of Ni_2_P doped CdS. The diffraction peak shift towards the higher angle for the doped material induces the expansion in the crystal lattice that increases the interplanar spacing. The XRD pattern of Pt/TiO_2_ shows diffraction peaks at 25.34°, 38.15°, 48.10° and 55.11° which was assigned to Pt/ TiO_2_. For bare TiO_2_, an intense peak at 25.30°, 37.79° and 48.03° corresponds to (101), (004) and (200) of the anatase phase of TiO_2_, respectively. In Pt/TiO_2_ a TiO_2_ has been detected at 47.44°. The absence of diffraction peak of the Pt on the Pt/TiO_2_ composite indicated that the platinum was well dispersed in TiO_2_. The high degree of dispersion was also confirmed by the HR-TEM image of the TiO_2_ and Pt/TiO_2_ in Fig. 2H, in which a clear lattice image of TiO_2_ and Pt particles on TiO_2_ are evident. The amount of doped Pt was less than 2.0% which is very less concentration to be detected by the XRD instrument significantly.

### SEM and HRTEM studies

**Figure 3:**
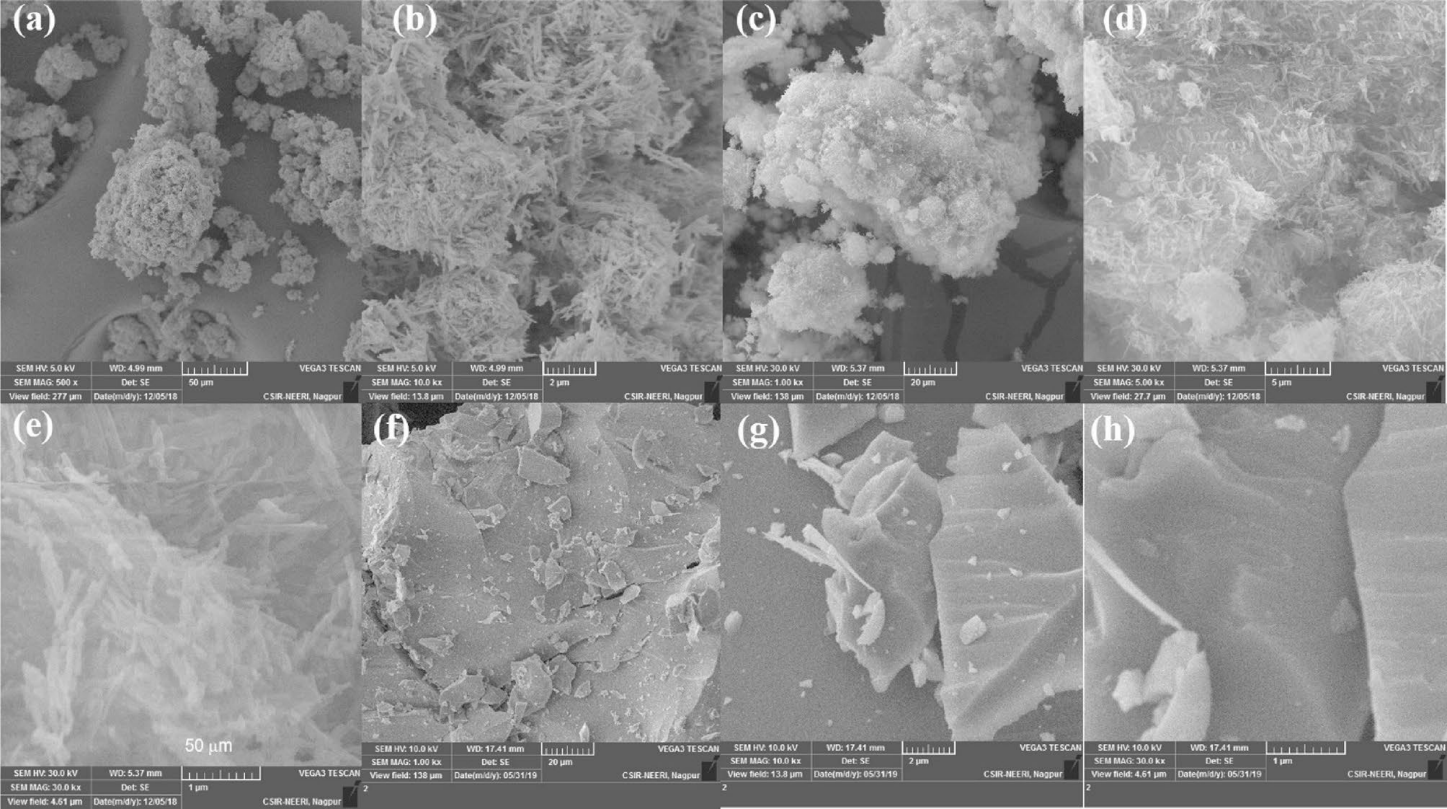
SEM images of Ni_2_P/CdS (**a**–**d)** and Pt/TiO_2_ (**e**–**h)**.

SEM and HRTEM analysis were carried out to investigate the morphology and microstructure of prepared photocatalysts. In Fig. 2a–d shows crystalline CdS nanorods with an average diameter of 100–5 nm while nickle phosphide deposited over CdS via solvothermal process showed similar geometry of nanorods and their diameter has not apparent change. The distance of lattice fringe of 0.33 and 0.22 nm for CdS and Ni_2_P, respectively, clearly shows that they are in close contact. The images clearly showed that the length of Ni_2_P/CdS is a little smaller than pure CdS while the structure and the results confirmed that morphology had no effect of the deposition of Ni_2_P on CdS. In Fig. 2e–h Pt/TiO_2_ crystal clearly shows platinum deposited on the TiO_2_ crystals which are seen as black spots at high resolution in TEM images^32^. In Fig. 3 SEM images of CdS (a–d) and Ni_2_P/CdS (e–h) from 200 to 1 μm indicates that there is no change after deposition of nickel on CdS nanorods, especially in d and h clearly showing that after and before deposition of Ni_2_P the nanorods structures are clearly observed in both images. SEM results corroborate the finding of TEM that deposition of co-catalysts like Ni_2_P and Pt nanostructure on CdS and TiO_2_ has minimal impact on the structural morphology of the materials.

### UV–Vis diffuse reflectance spectroscopic (DRS) studies

**Figure 4:**
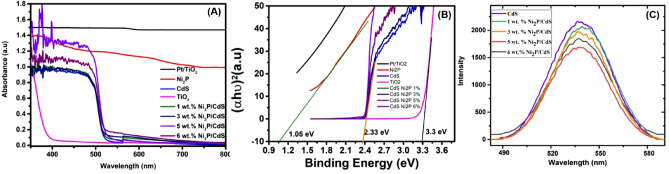
UV–Vis diffuse reflectance spectrum (**A**), Tauc plots (**B**) and photoluminescence spectrum of (**C**) of synthesized catalysts CdS, 1 wt% Ni_2_P/CdS, 3 wt% Ni_2_P/CdS, 3 wt% Ni_2_P/CdS, 5 wt% Ni_2_P/CdS and 6 wt% Ni_2_P/CdS.

The diffuse reflectance UV–Vis absorption spectra of synthesized composites were assembled for an area of 200–700 nm. The strong absorption peak ~ 264 to 296 nm indicates the interband transition between Ni_2_P–CdS and Pt–TiO_2_ (Fig. 4A). The absorption at a wavelength shorter than 390 and 580 nm in Ni_2_P/CdS and Pt/TiO_2_ can be ascribed as intrinsic bandgap absorption. These catalysts show the broad absorption in a visible range due to Ni_2_P and Pt deposition on CdS and TiO_2_^33^. The bandgap E_g_, of both the samples estimated using the Tauc’s relation given below in Eq. (2) is as follows;

2 $$\mathrm{\alpha h\upsilon }=\mathrm{ A }{(hv-Eg)}^{n}$$

where A is a constant, *Eg* the semiconductor bandgap and n is a number equal to 1/2 for the direct gap. The (αhυ)^2^ versus energy graphs were plotted. In Fig. 4, a straight line was fitted for the straight segment, this straight line was extrapolated to the X-axis to get the band gap values. The catalysts Ni_2_P, TiO_2_ and Ni_2_P/CdS have shown a bandgap of 1.05 eV, 3.3 eV and ~ 2.33 eV, respectively (Fig. 4B). The bandgap of CdS is shifted to left after loading of Ni_2_P and maximum shift has been observed in 5 wt% Ni_2_P/CdS indicating deposition of Ni_2_P on CdS and improved photocatalytic efficiency for CO_2_ reduction.

A series of experiments were conducted to understand the catalyst better. UV-DRS and photoluminescence (PL), was performed to investigate the photocatalytic mechanism for Ni_2_P/CdS composite. In the Fig. 4A, it is seen that CdS shows an absorption edge of approximately 490 nm^34^. The UV and visible light absorption intensity increased compared to pure CdS and 1–6 wt% Ni_2_P/CdS samples, which was attributed to the reduction of reflectivity^35^. The photoabsorption property has been increased by increasing the amount of Ni_2_P because of its dark colour. A slight redshift is seen in the samples of Ni_2_P/CdS and thus, the Ni_2_P is loaded onto the surface rather than the crystal lattice. In the present work, the optimal value was attained in 5 wt% Ni_2_P/CdS sample. In addition, as the diameter grows, it takes longer for the electrons to pass nanoparticles into the surface of Ni_2_P and less electrons to produce methanol. The maximum yield of methanol was obtained (2843 μmol/g cat) by using 5 wt% Ni_2_P/CdS catalyst under a visible light source of 300 W Xe lamp with 420 nm cutoff filter and 300 W tungsten light for 1 h. The Hg lamp with 300 W was the light source for Pt/TiO_2_ catalyst. As expected, increased Ni_2_P loading resulted in increased photoactivity. Ni_2_P served as an active centre for the production of methanol by electron trapping to decrease the recombination rate of electron–hole pairs during photocatalyst excitation. The adsorption of reactant CO_2_ can be supported by surface hydroxyl (OH) of CdS to improve photoreaction^36^. The amount of hydroxyl on the surface of CdS increased with increasing Ni_2_P loading, and the total CO_2_ photoreduction increased significantly. However, excess of Ni_2_P on the CdS surface (6 wt%-Ni_2_P/CdS), resulted in less catalyst light exposure. As a result, electron and hole pair photoexcitation was decreased because less photo-energy was absorbed. Therefore, an optimal Ni_2_P loading on the catalyst is a 5 wt% Ni_2_P and the highest methanol yields were found at this percentage under experimental conditions.

The photoluminescence (PL) is obtained from the radiative recombination of the photo-generated electron–hole pair. The (PL) emission experiment was carried out at an excitation wavelength of 350 nm. A series of studies includes loading of Ni_2_P on CdS (1 wt% Ni_2_P/CdS, 3 wt% Ni_2_P/CdS, 5 wt% Ni_2_P/CdS and 6 wt% Ni_2_P/CdS) as shown in Fig. 4C were restricted to the recombination of photoexcited electron–hole pairs. It appears that all samples exhibit a high emission at 535 nm because free electrons are radially recombined with free holes at the valence bands. Due to the increase in Ni_2_P concentration on CdS the PL strength compared to bare CdS was noticeably reduced. The lowest intensity was attained with a 5 wt% Ni_2_P/CdS composite which suggested that co-catalysts Ni_2_P was able to substantially reduce the population of single-excitons with the presence of an intimate interface of Ni_2_P and CdS composites.

### XPS spectra

**Figure 5:**
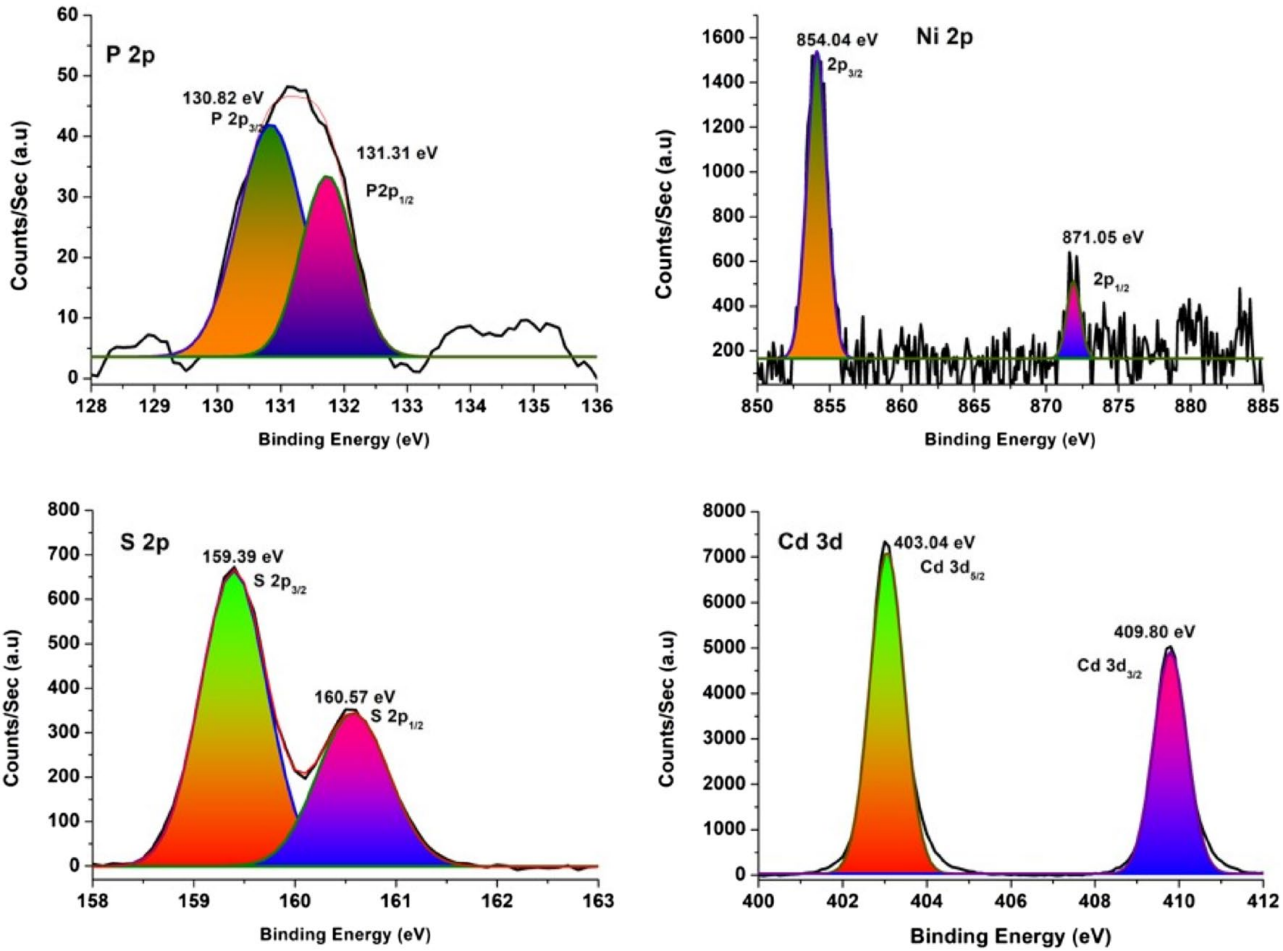
XPS spectra of Ni_2_P/CdS nanostructures, in images (**a**) Ni 2p, (**b**) S 2p, (**c**) P 2p and (**d**) Cd 3d.

The valence state of the elements and the chemical composition of Ni_2_P/CdS photocatalyst were studied through XPS measurements. Figure 5 represents the binding energy (BE) curve for the core level spectra of Cd 3d, S 2p, Ni 2p, and P 2p constituent elements in the photocatalyst. Figure 5a displays the core level spectrum of Cd 3d area where 403.04 eV and 408.80 eV peaks correspond to Cd 3d_5/2_ and Cd 3d_3/2_, respectively. These values are close to earlier reported values^37^. In Fig. 5b, S2p has two peaks positioned at 160.54 eV and 159.38 eV are corresponding to S 2p_1/2_ and S 2p_3/2_ orbitals of divalent sulfide ions (S^2−^), respectively, which are in line with the formation of CdS^38^. The characteristic binding energies of peaks located at 854.35 eV, 871.72 eV, 130.82 eV and 131.15 eV correspond to Ni 2p_3/2_, Ni 2p_5/2_, P 2p_3/2_ and P 2p_1/2_ respectively, shown in Fig. 5c,d^39,40^. These results clearly demonstrate the existence and strong interaction between Ni_2_P and CdS, which is in good agreement with the TEM analysis.

### Photocatalytic reduction of CO_2_ to methanol

**Scheme 2:**
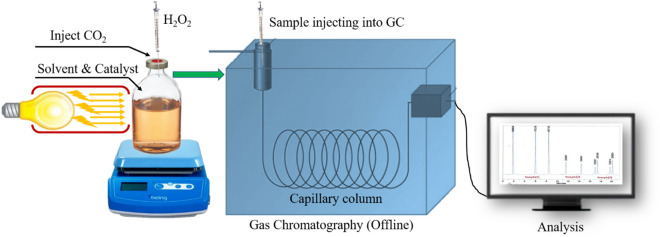
A diagrammatical representation of photocatalytic reduction of CO_2_ to methanol process.

A known amount of CO_2_ is purged into DMF after removal of moisture from the solvent with nitrogen purging at least for 30 min. 20 mg of the prepared photocatalyst was well dispersed in the mixture of DMF with TEA as a sacrificial electron donor. Carbon dioxide is added to the reaction mixture, followed by hydrogen peroxide and irradiated with light for the different time intervals. Liquid samples were filtered with a microsyringe filter and injected in offline GC to detect products (Scheme 2).

**Figure 6:**
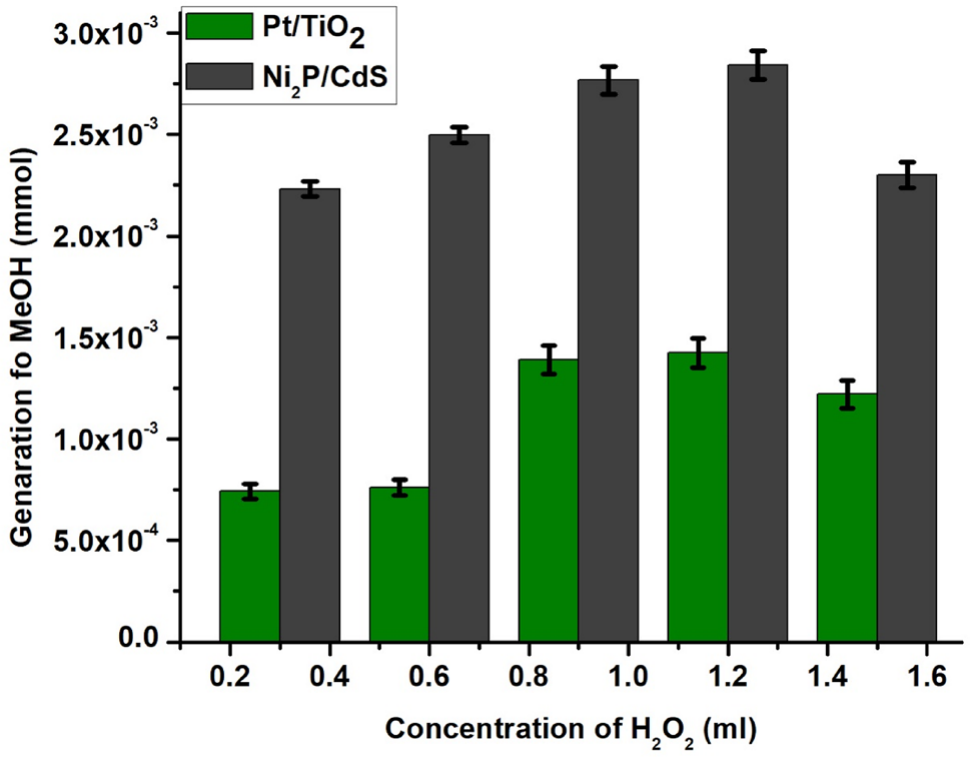
Effect of increasing concentration of H_2_O_2_ from 0.2 to 1.6 ml on the conversion of CO_2_ to MeOH generation.

The photocatalytic reduction of CO_2_ to methanol was performed for both prepared catalysts. The catalysis test results showed that Ni_2_P/CdS is highly active than Pt/TiO_2_ under the influence of light (Fig. 6). An increment in the concentration of H_2_O_2_ shows a positive effect on methanol generation until the concentration researches 1.5 ml. There is no detection of methanol over oxidation products like formaldehyde and formic acid etc., in the GC and GC–MS spectrum indicting high selectivity towards methanol. There is less variation in methanol formation between 1 and 1.5 ml of H_2_O_2_, indicating that the reaction is completed at this point. However, the additional increment in H_2_O_2_ concentration of more than 1.5 ml shows the reduction in the formation of methanol. This may be attributed to a decrease in the availability of CO_2_ in the reaction mixture.

**Figure 7:**
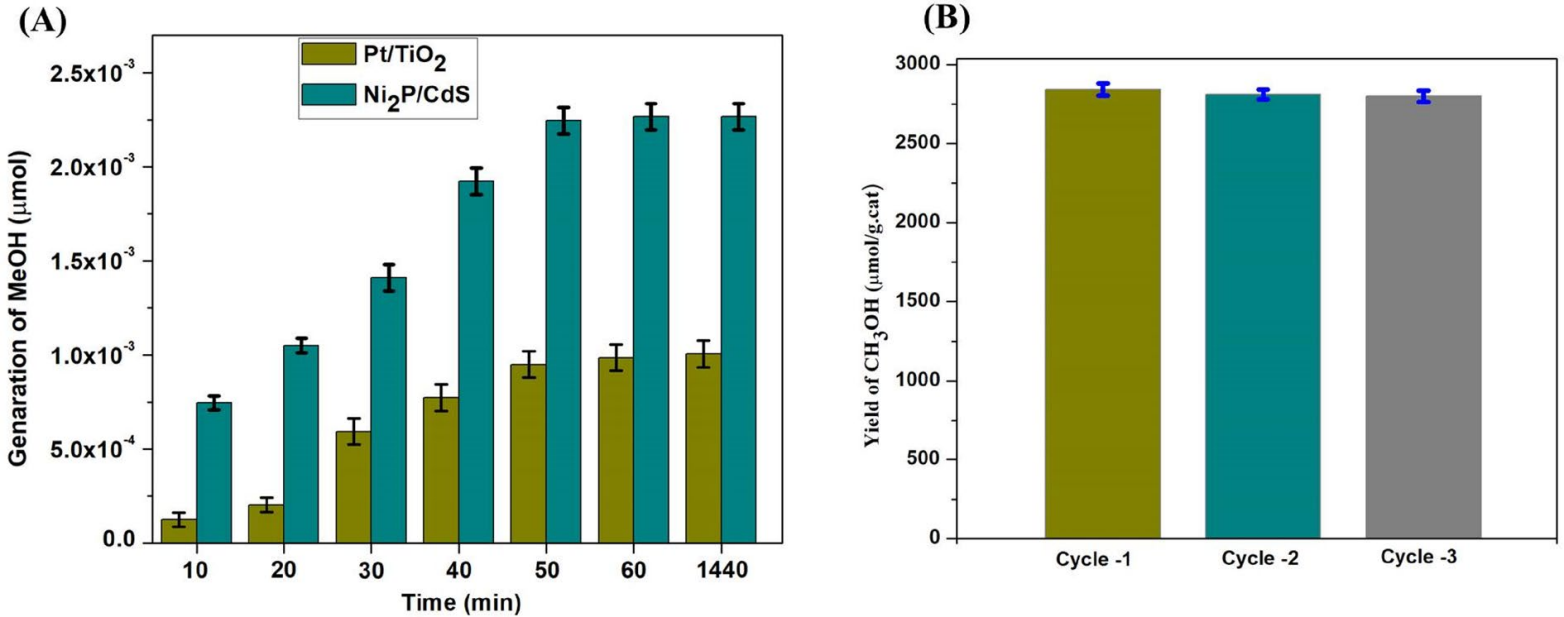
The plots against reaction time in minutes (10–1440) and methanol produced from the CO_2_ with catalyst Ni_2_P/CdS and Pt/TiO_2_ (**A**) and reproducibility of catalyst Ni_2_P/CdS for 3 cycles at 1 h time (**B**).

Therefore the ideal concentration of H_2_O_2_ for the reduction of CO_2_ is 1.5 ml/20 mg of catalyst. The effect of light over a different time is also done using the same catalyst. It is concluded that as the time duration increases the concentration of methanol increases. After an hour, no significant impact on the generation of methanol suggesting the catalyst can get exhausted (Fig. 7A). Nanocomposites typically have specific features that support the catalyst and are reusable. To illustrate this characteristic, we have studied the performance of selected catalyst 5 wt%-Ni_2_P/CdS to reduce CO_2_ to CH_3_OH. The obtained results were strongly supporting to the production of methanol after 3-cycle at 1 h time. After completion of 1 h reaction, the catalyst was removed from the reaction mix and dried and the next reaction was conducted. Based on the findings, the formation of CH_3_OH after each run does not effect a significant loss in efficiency of catalyst (Fig. 7B).

### Quantum efficiency for CO_2_ reduction

Photochemical efficiency describes the percent of absorbed photons that reduce CO_2_ to products. CO_2_ adsorption on photocatalysts affects the efficacy of photochemical efficiency. The quantum outcome of the reaction is widely called photochemical efficiency. The photo-reduction of CO_2_ to methanol requires two electrons, the photochemical efficiency of the reaction is obtained by the equation given below^41^.

Here we calculate the quantum efficiency at λ = 420 ± 15 nm. The catalyst mixture was irradiated by a 300 W Xe lamp for 1 h. The average incident irradiation was determined to be 2.75 W/cm^2^ and the area was 17.59 cm^2^. The amount of methanol produced in 1 h was 2843 μmol. All the calculations are given below.

The number of incident photons (N) in 1 h over 17.59 cm^2^ area:

$$N=\frac{E\uplambda }{hc}= \frac{2.75\times 17.59\times 3600\times 420\times {10}^{-9}}{6.626\times {10}^{-34}\times 3\times {10}^{8}}= 3.679405\times {10}^{20}$$

$${\text{QE}} = 6 \times \frac{{{\text{the}}\;{\text{number}}\;{\text{of}}\;{\text{product}}\;{\text{molecules}}\;{\text{produced}}}}{{{\text{the}}\;{\text{number}}\;{\text{of}}\;{\text{incident}}\;{\text{photons}}}}$$

$$=\frac{6\times 2843\times {10}^{-6}\times 6.02\times {10}^{23}}{3.679405\times {10}^{20}}=2843$$

Photochemical efficiency depends on the intensity and wavelength of radiation. According to experiment results, a maximum quantum yield of 27.91% was obtained by 5 wt% Ni_2_P/CdS (Table 1).

**Table 1:** Quantum yield obtained by catalysts **1–5.**

S. No	Catalyts	Yield	AQY
1	1 wt% Ni2P/CdS	542	5.32070756
2	3 wt% Ni2P/CdS	1623	15.9326723
**3**	**5 wt% Ni2P/CdS**	**2843**	**27.9091727**
4	6 wt% Ni2P/CdS	981	9.63028436
**5**	**Pt/TiO**_**2**_	**1425**	**13.9889452**

Bold is indicating present work which we are reporting in this manuscript, with significant yield.

### Mechanism

**Figure 8:**
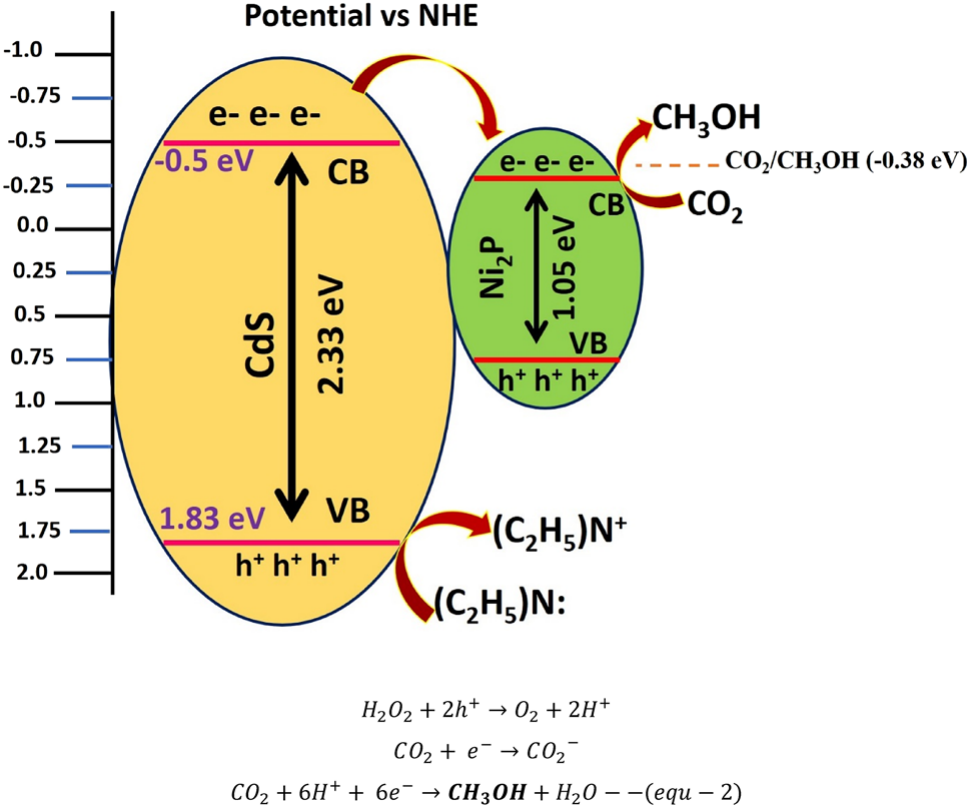
Probable mechanism of charge transfer and CO_2_ reduction by 5 wt%-Ni_2_P/CdS composite and chemical equations. Comparision of Ni_2_P/CdS and Pt/TiO_2_ for photoreduction of CO_2_ to methanol is provided in Table 2. The synthesised 5% Ni_2_P/CdS photocatalyst in this work appears to outperforming the other reported photocatalysts.

Based on the experimental results a plausible schematic mechanism basic the robust methanol production over 5 wt%-Ni_2_P/CdS composite was proposed (Fig. 8). It is well known that the CB edge of CdS is more negative than that of Ni_2_P. Thus on irradiation with visible light the photogenerated electrons can efficiently transfer from the conduction band (CB) of CdS to Ni_2_P. The electrons accumulating on the Ni_2_P particles can reduce CO_2_ into CO_2_^−^ while the holes on CdS can oxidize H_2_O_2_ to produce O_2_ and H^+^. Then CO_2_^−^ reacts with H^+^ to produce CH_3_OH in presence of sacrificial electron donor TEA (Eq. 2). The effective separation of the photo-generated electrons and holes in CdS further improves photocatalytic activity.

**Table 2:** Comparison of methanol yield over different photocatalysts.

Sr. no.	Photo-catalyst	MeOH (μmol/g.cat)	Solvent	References
1	CeO_2_-TiO_2_	19	NaOH	^42^
2	CuO/TiO_2_	27	KHCO_3_/Na_2_SO_3_	^43^
3	Ni@NiO/InTaO_4_-N	320	Water	^44^
4	CoPc-TiO_2_	1032	NaOH/Na_2_SO_3_	^45^
5	rGO-CuO124	1282	DMF/Water	^46^
6	Ruthenium Phosphine	2210	THF/EtOH/C_34_H_29_P_3_	^47^
7	Ru-CoPc@TiO_2_@SiO_2_@Fe_3_O_4_	2570	TEA/Water	^48^
8	Go-CoPc	3781	TEA/Water	^49^
9	rGO@CuZnO@Fe_3_O_4_	2656	DMF/Water	^50^
10	1 wt% Ni_2_P/CdS	542	DMF/TEA/H_2_O_2_	Current work
11	3 wt% Ni_2_P/CdS	1623	DMF/TEA/H_2_O_2_	Current work
**12**	**5 wt% Ni**_**2**_**P/CdS**	**2843**	**DMF/TEA/H**_**2**_**O**_**2**_	**Current work**
13	6 wt% Ni_2_P/CdS	981	DMF/TEA/H_2_O_2_	Current work
14	Pt/TiO_2_	1425	DMF/TEA/H_2_O_2_	Current work

Bold is indicating present work which we are reporting in this manuscript, with significant yield.

## Conclusion

The reduction of CO_2_ via visible light to methanol is achieved successfully. Here we are reporting methanol generation over noble metal-free hybrid semiconductor photocatalyst (Ni_2_P/CdS) under visible-light-driven CO_2_ reduction. The photocatalytic activity of the catalyst was enhanced by the homogenous dispersion of the co-catalyst. The synthesized Pt/TiO_2_ photocatalyst was adopted as the reference for comparison of the photocatalytic activity of the Ni_2_P/CdS nanocomposites under similar experimental conditions with an increase in Ni_2_P loading, the photocatalysts showed increased crystallinity. The CdS loaded with Ni_2_P showed greater efficiency than CdS for the formation of methanol from CO_2_. The 5 wt% loading of N_i2_P on CdS was found to be optimal among the different compositions and afforded the highest product yield (2843 μmol/g cat). The reaction was found to be completely photocatalytic, as in the absence of visible light, no conversion was observed. The synthesized photocatalyst was heterogeneous and showed clear 3 recycle runs with no change in catalytic efficiency and also no significant leaching and change in morphologywas observed. Also, the reduction rate of CO_2_ is much higher than the previously reported catalysts. These results may provide a new avenue for various photocatalytic applications.
